# Why sex matters

**DOI:** 10.7554/eLife.74935

**Published:** 2021-12-02

**Authors:** Josette J Wlaschin, Sangeetha Hareendran, Claire E Le Pichon

**Affiliations:** 1 Eunice Kennedy Shriver National Institute on Child Health and Human Development, National Institutes of Health Bethesda United States; 2 Department of Biology, Johns Hopkins University Baltimore United States

**Keywords:** microglia, spinal cord, pain, CSF1, meninges, Treg, Mouse

## Abstract

The immune mechanisms underlying hypersensitivity to pain after nerve injury are different in male and female mice.

**Related research article** Kuhn JA, Vainchtein ID, Braz J, Hamel K, Bernstein M, Craik V, Dahlgren MW, Ortiz-Carpena J, Molofsky AB, Molofsky AV, Basbaum AI. 2021. Regulatory T-cells inhibit microglia-induced pain hypersensitivity in female mice. *eLife* **10**:e69056. doi: 10.7554/eLife.69056

Pain cautions our bodies against harmful stimuli – such as a burning flame or the pointy end of a needle – and protects us when we are injured. These stimuli are detected by sensory neurons, which transmit signals to the spinal cord and brain. Damaging these neurons can lead to persistent and chronic pain, but the mechanisms underlying this are not fully understood.

One important player in controlling pain related to nerve damage is the immune system (Calvo et al., 2012; Scholz and Woolf, 2007). Previous work showed that injured sensory neurons release a protein called CSF1 (short for colony stimulating factor 1), which activates microglia, the main immune cell type in the brain and spinal cord. In this activated state, microglia proliferate, change their form and alter their behavior.

In 2016, a group of scientists discovered that male mice became hypersensitive to touch when their microglia were activated by nerve injury or by injecting CSF1 in to the space around the spinal cord (Guan et al., 2016). However, microglia have also been shown to be sexually dimorphic, playing different roles in disease and pain in males and females (Mogil, 2020). Now, in eLife, Allan Basbaum, Anna Molovsky and colleagues from the University of California, San Francisco – including Julia Kuhn and Ilia Vainchtein as co-first authors – report that microglia and another immune cell population respond differently to pain signals in male and female mice (Kuhn et al., 2021).

To investigate the mechanisms underlying hypersensitivity to touch, the team (which includes some of the researchers involved in the 2016 study) damaged the sciatic nerves of male and female mice lacking the gene for the CSF1 protein in their sensory neurons. Pain was assessed using the Von Frey assay, where mice are placed on an elevated grate and their paws are poked with different sized filaments (Decosterd and Woolf, 2000; Shields et al., 2003). Thick filaments will evoke a pain response that causes the mouse to flinch and withdraw its paw; whereas, thinner filaments only elicit this response when mice are hypersensitive to touch.

As shown previously, male mice deficient in CSF1 were not hypersensitive to touch after nerve injury. Female mice lacking CSF1, however, still withdrew their paws when poked with thinner filaments, suggesting that the mechanism underlying hypersensitivity in females is different to males. To confirm these findings, Kuhn et al. injected CSF1 near the spinal cord and assessed pain in the absence of nerve injury. As expected based on the previous results, the male mice became hypersensitive to touch, whereas the females did not (Figure 1). Further experiments examining the genes expressed by microglia after injection of CSF1 revealed that male mice upregulated different genes compared to females, including genes associated with disease, and the activation and recruitment of immune cells.

**Figure 1.**
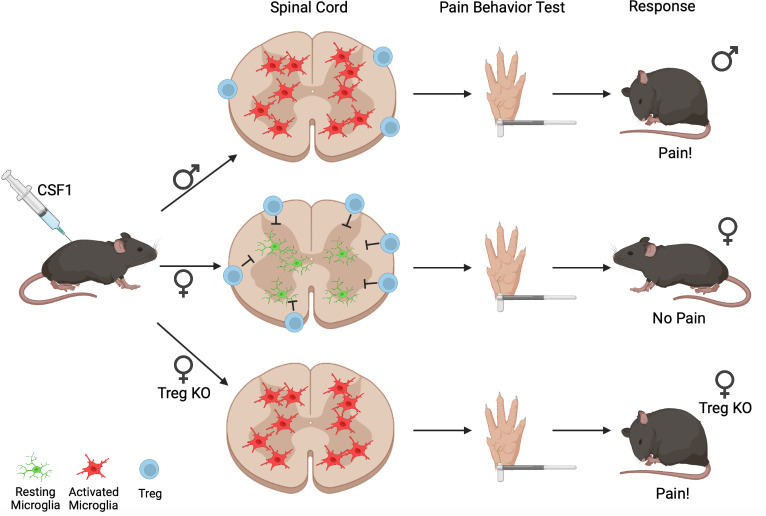
The differing effects of CSF1 injection on male and female mice. When CSF1 is injected into wild-type mice, microglia in the spinal cord become activated (red cells) in male mice (top) but not females (middle). In females, regulatory T-cells (Tregs, blue circles) present in the membrane layers surrounding the spinal cord block CSF1 from activating microglia, which remain in the resting state (green cells); when regulatory T-cells are depleted (Treg KO; bottom), the microglia of female mice respond to CSF1 the same way as in males (bottom). During Von Frey pain assessment tests, female mice with depleted levels of regulatory T-cells and male mice exhibit the paw withdrawal response typical of hypersensitivity (top and bottom); however, female mice do not elicit a hypersensitive pain response. This indicates that regulatory T-cells suppress the activation of microglia and development of a pain response after CSF1 injection, but only in female mice.

Image credit: Figure created using Biorender.com.

Other types of immune cells are known to influence how the central nervous system works under both normal and diseased conditions. To see if any of these might be involved in female pain sensation, Kuhn et al. examined which immune cells were present in the membrane layers surrounding the spinal cords of mice injected with CSF1. Females were found to have more regulatory T-cells, which are potent inflammation suppressors. Kuhn et al. wondered if having a greater number of regulatory T-cells counteracts the effects of CSF1, so they repeated the experiments in female mice in which regulatory T-cells had been depleted. This revealed that without regulatory T-cells, female mice also develop hypersensitivity after CSF1 injection, and their microglia express a more similar pattern of genes to the microglia of males (Figure 1).

This study demonstrates that the immune system plays different roles in the pain pathways of male and female mice after nerve injury. In male mice, microglia are the major immune cell type driving pain induced by CSF1 injection, while regulatory T-cells repress this pathway in females. This work highlights the need to include males and females in scientific research, and the importance of considering sex-specific approaches for pain management. It also opens up interesting questions for future investigation. For example, it is unclear how regulatory T-cells are recruited in females after CSF1 injection, and the mechanisms underlying pain hypersensitivity in female mice remain to be discovered.
